# Single incision laparoscopic adjustable gastric band: technique, feasibility, safety and learning curve

**DOI:** 10.1308/003588413X13511609954978

**Published:** 2013-03

**Authors:** AJ Osborne, R Clancy, GWB Clark, C Wong

**Affiliations:** ^1^North Bristol NHS Trust, UK; ^2^Cardiff and Vale University Health Board, UK

**Keywords:** Laparoscopic surgery, Obesity surgery, Single incision

## Abstract

**Introduction:**

Single incision laparoscopic surgery (SILS) is established in many procedures but not in bariatric surgery. One explanation may be that SILS is technically demanding in morbidly obese patients. This report describes our technique and experience with single incision laparoscopic adjustable gastric banding (SILAGB).

**Methods:**

Prospective data collection was performed on consecutive obese patients who underwent SILAGB between November 2009 and February 2011. A single 3cm transverse incision in the right upper quadrant was used for a Covidien SILS^™^ multichannel access port. The technique is described with a standard pars flaccida approach and the ‘tips and tricks’ needed for a wide range of candidates using standard laparoscopic equipment.

**Results:**

A total of 29 patients (27 female) with a median body mass index of 41kg/m^2^ (range: 35–52kg/m^2^) and median age of 44 years (range: 22–57 years) underwent SILAGB. There were no ‘conversions’ to a standard laparoscopic technique. Two cases required the addition of one single 5mm port. The only complications were two postoperative wound infections (one with a port site infection requiring replacement of the port) and one faulty band requiring replacement. There were therefore two returns to theatre and no 30-day deaths. All patients were discharged on the first postoperative day. In this series, operative times reduced significantly to be comparable with the conventional laparoscopic approach.

**Conclusions:**

SILAGB is safe and feasible in the morbidly obese. Proficiency in this technique using conventional laparoscopic equipment can be achieved with a short learning curve.

Bariatric surgery is currently the only effective means of achieving clinically significant long-term weight loss in the morbidly obese where rapid mobilisation and enhanced recovery after surgery is a particular advantage.^1^ Case reports for single incision laparoscopic adjustable gastric banding (SILAGB) surgery have been published since 2008.^2^ However, single incision laparoscopic surgery (SILS) for obesity has been slow to increase in popularity compared with other procedures such as cholecystectomy, where it is more established.^3^ The reason for the slow uptake in SILAGB may be that it is perceived as technically demanding, resource consuming and likely to have a significant learning curve in the obese patient. This report describes our technique and experience with SILAGB.

## Methods

A prospective electronic database was maintained for all SILAGB performed by the senior author from November 2009. Consecutive patients requiring obesity surgery were considered for SILAGB; there were no specific exclusion criteria.

### Surgical technique

The patient is placed supine under general anaesthesia. The operator is positioned on the patient’s right side with the scopist positioned either between the patient’s split legs or simply behind the operator on the patient’s right side.

A 25–30mm transverse incision is made 5cm below the xiphisternum to the right of the midline and falciform ligament. The subcutaneous fat is separated and the anterior rectus sheath incised, exposing the rectus muscle. This is retracted laterally and the posterior rectus sheath opened to gain access into the abdominal cavity. A prepared gastric band is placed inside the abdomen at this stage before inserting a SILS™ port (Covidien, Mansfield, MA, US).

**Figure 1:**
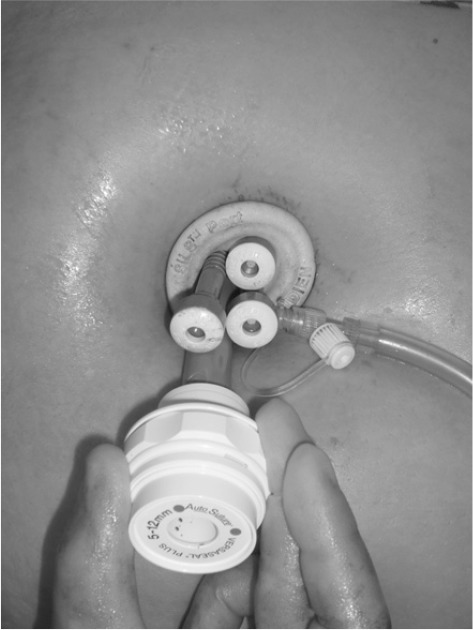
The single port used for the laparoscopic adjustable gastric banding

**Figure 2:**
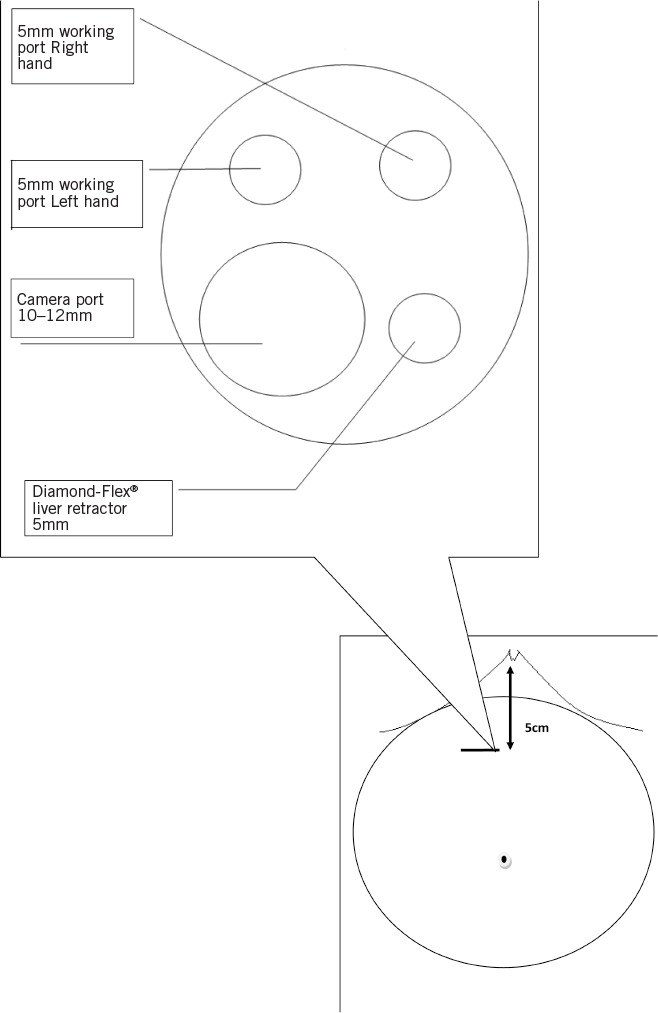
Diagrammatic representation of the single incision laparoscopic adjustable gastric band procedure

The port is positioned such that the insufflation tube is at 5 o’clock (Fig 1). The insufflation tubing is replaced with a 5mm SILS™ port cannula equipped with the insufflation attachment. Pneumoperitoneum is established to 15mmHg. Two operating ports using the 5mm SILS™ port cannulas are inserted at the 1 o’clock and 11 o’clock positions (1 o’clock – right hand; 11 o’clock – left hand). A 12mm SILS™ cannula is inserted in the remaining hole at 7 o’clock for a 10mm 30º laparoscope (Fig 2). A 5mm Diamond-Flex^®^ liver retractor (CareFusion, Waukegan, IL, US) is inserted through the insufflation access port. The liver retractor will also elevate the falciform ligament, allowing good views at a comfortable distance from the target. Standard laparoscopic instruments are used.

A normal pars flaccida approach with gastrogastric tunnelling allows placement of a Bioring^®^ gastric band (Cousin Biotech, Werviq-Sud, France). Briefly, the pars flaccida is opened with a hook and the right crus identified. The peritoneum over the angle of His is also opened with the hook. A Goldfinger™ retractor (Ethicon Endo-Surgery, Cincinnati, OH, US) is passed retrogastrically to allow positioning of the gastric band around the gastric pouch. Size 0 Ethibond^®^ sutures (Ethicon, Somerville, NJ, US) are passed into the abdomen via the 12mm port.

A gastrogastric wrap is created with interrupted size 0 Ethibond^®^ sutures by extracorporeal knotting. When a hiatus hernia is encountered, the hernia sac is dissected anteriorly from the crura and the hiatus closed with size 0 Ethibond^®^. The gastric band tubing is retrieved and the port removed before the anterior rectus sheath is closed with loop PDS^®^ sutures (Ethicon). A subcutaneous pocket is created in the left upper quadrant for securing the gastric band access port.

The approach from the patient’s right allows direct vision of the retrogastric area, thereby avoiding any blind dissection from lack of triangulation if approached from the umbilicus. To avoid clashing and crowding of instruments (swording), tissues are grasped or retracted a greater distance away from the target than in the usual technique in multiport laparoscopic surgery. Once the laparoscope is in the correct position to achieve the view, the laparoscope is rotated rather than repositioned in order to prevent swording.

## Results

There were 29 patients with a median body mass index (BMI) of 41kg/m^2^ (range: 35–52kg/m^2^). Of these, 27 were female and the mean age was 45 years (SD: 10 years, range: 22–63 years). There were no ‘conversions’ to a standard multiport laparoscopic technique but two cases early in the series required the addition of one single 5mm port owing to a bulky liver. Four patients required additional crural repair for a hiatus hernia. The complications were two postoperative wound infections: one with a port site infection requiring replacement of the port (following this, the technique was altered to move the port from the original incision to a subcutaneous pocket created in the left upper quadrant) and one faulty band requiring replacement. There were therefore two returns to theatre and no 30-day deaths. All patients were admitted overnight and discharged on the first post-operative day.

**Figure 3:**
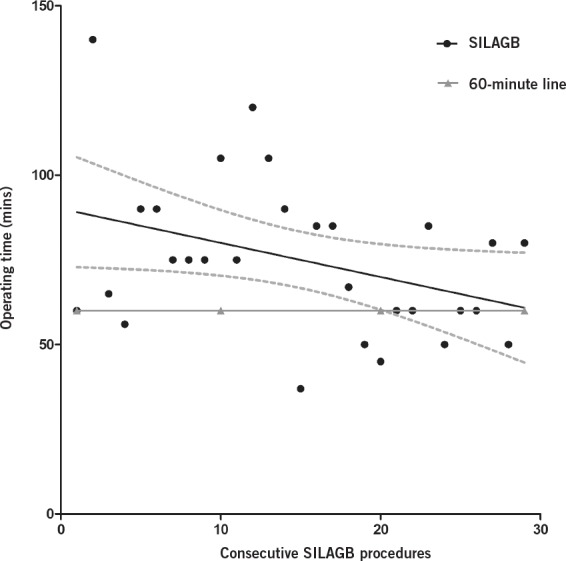
The learning curve for single incision laparoscopic adjustable gastric banding (SILAGB); 95% confidence intervals can be seen as dotted lines

The operating time reduced significantly in this short series (Fig 3). The Pearson correlation coefficient (r) was -0.4 (95% confidence interval: -0.649–-0.005) with a two-tailed *p*-value of <0.05. The confidence intervals can be seen in Figure 3. This shows that after 29 patients, the operative time was less than 60 minutes.

## Discussion

The reports of SILS are increasing. A review in 2011 by Huang reported 11 series (case reports were excluded) of single incision laparoscopic bariatric surgery, including gastric banding, sleeve gastrectomy and gastric bypass.^4^ In this review, 14% of patients required an additional port for a liver retractor or a further working port site. Only one complication of wound infection was reported in these series. Our results are comparable with the established literature, suggesting SILAGB can be performed safely in morbid obesity surgery with an ‘all comers’ approach. The learning curve is relatively short with the regression line for operative time falling to 60 minutes after 29 patients. This is similar to other reports of operating times of 45–65 minutes and a learning curve of 35 cases.^5^

It has been suggested that when the BMI is over 45kg/ m2, SILAGB is more challenging.^4^ However, Cheregi *et al* found their mean operating time to be 61 minutes with no correlation between a higher BMI and an increased operating time.^6^ Our experience supports this.

Concomitant hiatal hernia can be repaired successfully during SILAGB as per standard repair for gastric band insertion. This is an independent significant predictor of additional operating time.^6^ In our series, we encountered four patients (14%) with a hiatus hernia. These were repaired easily with anterior closure of the hiatus.

Factors that enhance recovery such as early ambulation and good control of post-operative pain are important in reducing the risk of thromboembolic complications in this group of patients. SILAGB is reported to have significantly less post-operative pain than the standard laparoscopic approach while providing an improved cosmetic outcome.^7,8^ Patel *et al* suggested, however, that this advantage of less post-operative pain is lost in the event of an increased operation time.^7^ We agree that an additional port should be considered if the operative time is greater than 60 minutes. Our results suggest this will be important during the short learning curve when operating times are more likely to be over 60 minutes. We found this most helpful to consider when the patient had an especially bulky liver and a single 5mm port was placed in the left upper quadrant.

The very nature of bariatric patients with an abundance of visceral fat, subcutaneous fat and multiple co-morbidities poses a unique challenge for surgeons to perform surgery with a single incision.^4^ The choice of port is important as maintenance of the intra-abdominal pressure is key to this procedure. We have found the SILS™ multichannel access port is sufficient to maintain a seal even in the presence of very deep subcutaneous tissue. Crowding and clashing of equipment are inherent problems with SILS procedures. To avoid this, specialist equipment has been developed but at a higher expense. Nevertheless, when the price of the SILS™ multichannel system is compared with the four standard ports used in multiport surgery, the economic result is at least cost neutral (depending on local arrangements). Therefore, by using standard laparoscopic equipment as described here, we have found it possible to avoid any increase in cost.

We use standard laparoscopic equipment and a 10mm 30º laparoscope. The advantages of the standard scope include better light levels and the fact that the larger scope is more robust. In the presence of a large amount of intra-abdominal fat, swording can be reduced by grasping tissue further away from the target than normal while still achieving equally good exposure. Initially, we placed the gastric band access port directly in the 3cm incision. This resulted in one port infection. After creating a fresh subcutaneous pocket for the access port, no further problems were seen in this series.

Randomised control studies are required to evaluate the advantages of a single incision approach over the traditional multiport laparoscopic approach for adjustable gastric banding. However, surgeons need to be familiar with the technical aspects of SILAGB before such trials can take place.

## Conclusions

SILAGB is safe and feasible in morbidly obese patients. Proficiency in this technique using conventional laparoscopic equipment can be achieved with a short learning curve.

## References

[1] Snyder B , Wilson T , Mehta S *et al* Past, present, and future: critical analysis of use of gastric bands in obese patients. Diabetes Metab Syndr Obes 2010; 3: 55–65 21437077 10.2147/dmsott.s6935 PMC3047987

[2] Oltmann SC , Rivas H , Varela E *et al* Single-incision laparoscopic surgery: case report of SILS adjustable gastric banding. Surg Obes Relat Dis 2009; 5: 362–364 19460675 10.1016/j.soard.2009.03.003

[3] Romanelli JR , Earle DB . Single-port laparoscopic surgery: an overview. Surg Endosc 2009; 23: 1,419–1,427 19347400 10.1007/s00464-009-0463-x

[4] Huang CK . Single-incision laparoscopic bariatric surgery. J Minim Access Surg 2011; 7: 99–103 21197253 10.4103/0972-9941.72397 PMC3002018

[5] Galvani CA , Gallo AS , Gorodner MV . Single-incision and dual-incision laparoscopic adjustable gastric band: evaluation of initial experience. Surg Obes Relat Dis 2012; 8: 194–200 21126924 10.1016/j.soard.2010.09.017

[6] Cheregi JR , Tiesenga F , Torquati A , Lutfi R . Initial learning experience of laparoendoscopic single site (LESS) gastric banding: finding predictors of success. Obes Surg 2012; 22: 433–436 22116594 10.1007/s11695-011-0552-5

[7] Patel AG , Murgatroyd B , Ashton WD . Single incision laparoscopic adjustable gastric banding: 111 cases. Surg Obes Relat Dis 2011 7 13 [Epub ahead of print.] 10.1016/j.soard.2011.06.013 21890429

[8] Saber AA , El-Ghazaly TH , Elain A , Dewoolkar AV . Single-incision laparoscopic placement of an adjustable gastric band versus conventional multiport laparoscopic gastric banding: a comparative study. Am Surg 2010; 76: 1,328–1,332 21265344

